# MRI-Based Radiomics Nomogram for Selecting Ovarian Preservation Treatment in Patients With Early-Stage Endometrial Cancer

**DOI:** 10.3389/fonc.2021.730281

**Published:** 2021-09-09

**Authors:** Bi Cong Yan, Xiao Liang Ma, Ying Li, Shao Feng Duan, Guo Fu Zhang, Jin Wei Qiang

**Affiliations:** ^1^Department of Radiology, Jinshan Hospital, Fudan University, Shanghai, China; ^2^Department of Diagnostic and Interventional Radiology, Shanghai Jiao Tong University Affiliated Sixth People’s Hospital, Shanghai, China; ^3^Precision Health Institution, GE Healthcare, Shanghai, China; ^4^Department of Radiology, Obstetrics & Gynecology Hospital, Fudan University, Shanghai, China

**Keywords:** radiomics, nomogram, endometrial cancer, myometrial invasion, ovarian preservation

## Abstract

**Background:**

Ovarian preservation treatment (OPT) was recommended in young women with early-stage endometrial cancer [superficial myometrial invasion (MI) and grades (G) 1/2-endometrioid adenocarcinoma (EEC)]. A radiomics nomogram was developed to assist radiologists in assessing the depth of MI and in selecting eligible patients for OPT.

**Methods:**

From February 2014 to May 2021, 209 G 1/2-EEC patients younger than 45 years (mean 39 ± 4.3 years) were included. Of them, 104 retrospective patients were enrolled in the primary group, and 105 prospective patients were enrolled in the validation group. The radiomics features were extracted based on multi-parametric magnetic resonance imaging, and the least absolute shrinkage and selection operator algorithm was applied to reduce the dimensionality of the data and select the radiomics features that correlated with the depth of MI in G 1/2-EEC patients. A radiomics nomogram for evaluating the depth of MI was developed by combing the selected radiomics features with the cancer antigen 125 and tumor size. Receiver operating characteristic (ROC) curves were used to evaluate the diagnostic performance of the radiomics nomogram and of radiologists without and with the aid of the radiomics nomogram. The net reclassification index (NRI) and total integrated discrimination index (IDI) based on the total included patients to assess the clinical benefit of radiologists with the radiomics nomogram were calculated.

**Results:**

In the primary group, for evaluating the depth of MI, the AUCs were 0.96 for the radiomics nomogram; 0.80 and 0.86 for radiologists 1 and 2 without the aid of the nomogram, respectively; and 0.98 and 0.98 for radiologists 1 and 2 with the aid of the nomogram, respectively. In the validation group, the AUCs were 0.88 for the radiomics nomogram; 0.82 and 0.83 for radiologists 1 and 2 without the aid of the nomogram, respectively; and 0.94 and 0.94 for radiologists 1 and 2 with the aid of the nomogram, respectively. The yielded NRI and IDI values were 0.29 and 0.43 for radiologist 1 and 0.23 and 0.37 for radiologist 2, respectively.

**Conclusions:**

The radiomics nomogram outperformed radiologists and could help radiologists in assessing the depth of MI and selecting eligible OPTs in G 1/2-EEC patients.

## Introduction

Endometrial cancer (EC) is the most common gynecological cancer in developed countries (1). Approximately 11% of EC patients are diagnosed before the age of 50, and 5% are diagnosed before the age of 40 (2). Staging surgery (including hysterectomy and bilateral salpingo-oophorectomy [BSO]) is the primary treatment for EC. Adequate preoperative staging and triage are essential for determining the surgical procedures and adjuvant therapy.

However, BSO results in the abrupt disruption of hormone levels, with short-term intense menopausal symptoms that can compromise one’s quality of life and lead to osteoporosis, metabolic syndrome, and cardiovascular disease (3, 4). Studies have suggested that BSO and ovarian preservation treatment (OPT) have similar mortality in young women with early-stage EC and that OPT does not decrease the overall survival of these patients (5–7). Furthermore, young patients with early-stage EC are more likely to die from cardiovascular diseases than from EC (5). Thus, the decision to preserve the ovaries in young EC patients is critical. More recently, studies recommended that OPT can be considered for patients younger than 45 years with early-stage EC [grade 1 and 2 (G 1/2) endometrioid adenocarcinoma (EEC), and myometrial invasion (MI) < 50%] and without ovarian mass (8–10).

Dilatation and curettage (D&C) and magnetic resonance imaging (MRI) are two recommended ways to preoperatively evaluate the tumor grade and EC staging (11, 12). Radiomics is an emerging technology that correlates image-based features with clinically relevant oncological outcomes. Studies suggest that quantitative radiomics or texture features may be useful for evaluating the deep myometrial invasion (DMI), with a similar accuracy of 84.8% compared with the subjective interpretation by experienced radiologists (13, 14). Moreover, tumor size or volume as determined on MRI is also useful for evaluating the depth of MI (11, 15). The radiomics nomogram includes a numerical probability of important clinical tumor diagnostic information and is considered a useful tool for quantifying tumor risk factors (16–18).

Therefore, in this study, we developed an MRI radiomics nomogram to assist radiologists in selecting eligible candidates for OPT by assessing DMI in G 1/2-EEC patients younger than 45 years.

## Materials and Methods

### Patients

The Institutional Review Board approved this study (approval number 2020-10). Informed consent was obtained. From February 2014 to September 2019, the electronic medical records of a total of 135 consecutive histopathologically proven EC patients under 45 years of age were reviewed.

**Figure 1:**
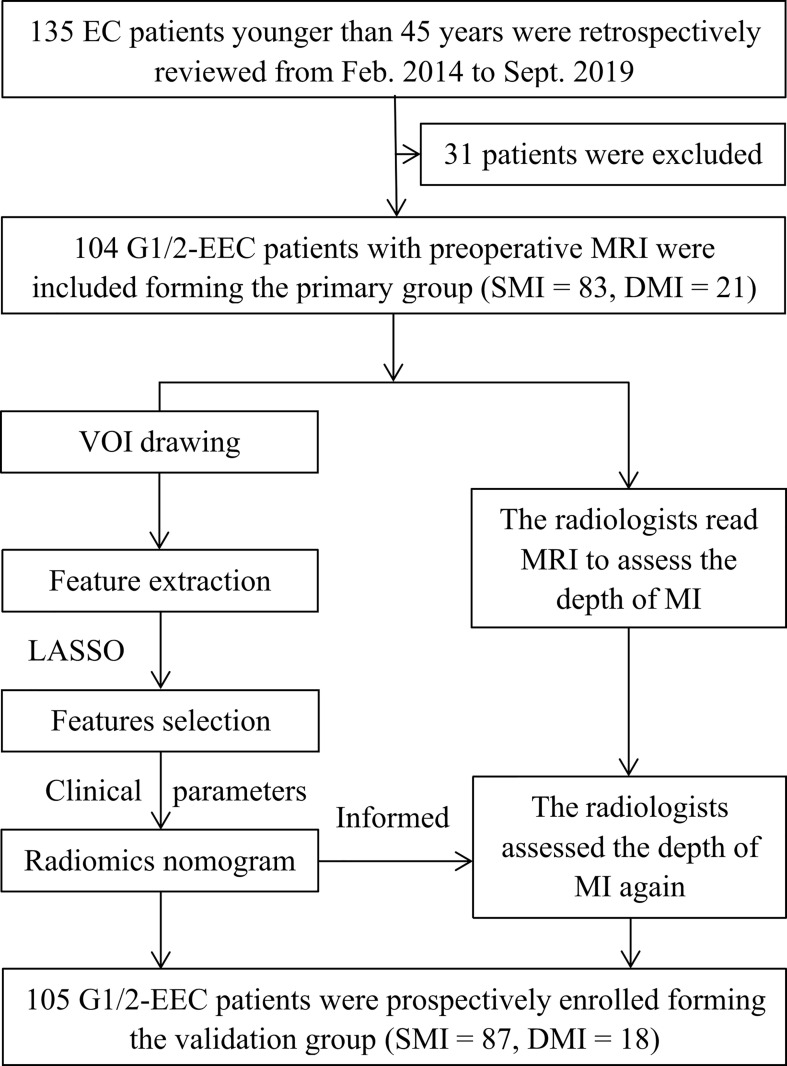
The workflow of this study. G 1/2, grade 1 and 2; DMI, deep myometrial invasion; EC, endometrial cancer; EEC, endometrioid adenocarcinoma; SMI, superficial myometrial invasion; MI, myometrial invasion; LASSO, least absolute shrinkage and selection operator.

Potential candidates for this study met the following criteria: 1) premenopausal patients younger than 45 years; 2) patients who underwent total hysterectomy with BSO and were histopathologically diagnosed with G1/2 and early-stage (International Federation of Gynecology and Obstetrics [FIGO] stages I–II) EEC; 3) patients without other or previous malignancies in the reproductive system, without family history of breast cancer, ovarian cancer, or Lynch syndrome; and 4) patients who underwent MRI scanning with sequences of T2-weighted imaging (T2WI), contrast-enhanced T1-weighted imaging (CE-T1WI), diffusion-weighted imaging (DWI), and apparent diffusion coefficient (ADC) maps. The exclusion criteria were as follows: 1) receiving anticancer treatment before surgery (n = 1); 2) absence of preoperative pelvic MRI (n = 9); 3) poor imaging quality due to artifacts or tumor invisible on MRI (n = 3); 4) no total hysterectomy performed (n = 5); 5) patients diagnosed with G3 EEC or non-EEC, or having a history of other cancer (n = 1); or 6) without serum cancer antigen 125 (CA125) information (n = 12). Furthermore, from September 2019 to May 2021, another 105 eligible EEC out of 109 patients were prospectively enrolled and formed the validation group. **Figure 1** shows the workflow of this study.

General clinical information, including age and tumor size (tumor maximum diameter measured retrospectively on T2WI), and CA125 from preoperative information, ER (estrogen receptor), PR (progesterone receptor), Ki67, and CK7 from postoperative pathology were collected. The univariate and multivariate logistic regression analyses were performed to analyze the predictive factors for DMI in G 1/2-EECs for OPT. The tumor stage was determined according to the 2014 FIGO staging system based on the final pathologic reports.

### Imaging

All enrolled patients received pelvic MRI within 30 days before surgery. The mean interval between MRI and surgery was 20 days (range, 5–26 days). MRI was performed using 1.5-T scanners (Avanto, Siemens, Erlangen, Germany) with an eight-channel pelvic phased-array coil. The patients lay supine and breathed freely during acquisition. The following sequences were obtained with a field of view (FOV) of 360 × 280 mm; axial T1WI (time of repetition [TR]/time of echo [TE] = 761/10 ms, matrix = 256 × 256, thickness = 4 mm); axial T2WI (TR/TE = 4,000/98, matrix = 256 × 256, thickness = 4 mm) with and without fat saturation (FS), coronal T2WI (FOV 400 mm, TR/TE = 3849/83 ms, matrix 320 × 256, thickness = 4 mm, slice gap = 1 mm), and sagittal T2WI (FOV 270 mm, TR/TE = 4,490/83 ms, matrix 320 × 256, thickness = 4 mm, slice gap = 1 mm); axial DWI (TR/TE = 4,000/100 ms, b = 0, 1,000 s/mm^2^, matrix = 128 × 128, thickness = 5 mm); and axial CE-T1WI with FS (TR/TE = 196/2.9 ms, matrix = 128 × 128, thickness = 4 mm) and sagittal CE-T1WI with FS (FOV 400 mm, TR/TE = 439/10 ms, thickness = 4 mm, matrix 320 × 256). CE-T1WI with FS was performed at the arterial phase (30–40 s), venous phase (75–90 s), and delayed phase (120–180 s) after the intravenous administration of gadopentetate dimeglumine (0.5 mmol/ml, GE Healthcare, Shanghai, China) at a dose of 0.2 mmol/kg of body weight and a rate of 2 to 3 ml/s. An ADC map was automatically generated based on DWI (b = 0 and b = 1000 s/mm^2^).

### Radiomics Feature Extraction

The patients’ images were first imported into MITK Workbench software (http://mitk.org/wiki/The_Medical_Imaging_Interaction_Toolkit_ (MITK)). The multisequence images from axial DWI, ADC map, and CE-T1WI-FS (delay phase) were subsequently aligned to axial T2WI images. Regions of interest (ROIs) were manually drawn by radiologist 1 (with 5 years of MRI experience in gynecological imaging) along the tumor margin on each T2WI slice and automatically matched to T1WI, DWI (1,000 s/mm^2^), ADC map, and CE sequences. After tumor segmentation, a 3D volume of interest (VOI) was obtained by resampling the image with sitkBSpline interpolator. The radiomics features were extracted using Pyradiomics (https://pypi.org/project/pyradiomics/). Imaging preprocessing was performed to ensure comparability of MRI gray values, and a fixed bin width of 1 was used to compute textural features. All radiomics feature implementations followed the IBSI recommendation (https://arxiv.org/abs/1612.07003).

### Feature Selection

**Figure 2:**
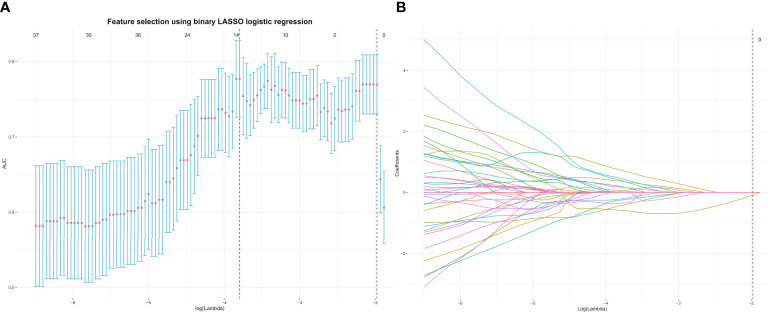
The top 13 radiomics features associated with DMI in G 1/2-EEC patients younger than 45 years were identified using the least absolute shrinkage and selection operator (LASSO) method in the primary group. **(A)** The parameter lambda (λ) is chosen using 10-fold cross-validation. **(B)** LASSO coefficient profiles of the selected features. A vertical line is plotted at the optimal value, resulting in 13 features with nonzero coefficients. EEC, endometrioid adenocarcinoma; DMI, deep myometrial invasion.

One month later, 50 patients were randomly chosen, and tumors were drawn by radiologist 1 repeatedly and by radiologist 2 (with 10 years of MRI experience in gynecological imaging) independently. The interclass and intraclass correlation coefficients (ICCs) of the extracted features were calculated to assess the reproducibility of radiomics features. We devised a three-step procedure for dimensionality reduction and selection of robust features (19). Firstly, features with both interclass and intraclass ICCs larger than 0.75 were considered robust and reproducible (20). Secondly, Pearson’s correlation was used to identify redundant features and the feature was selected for subsequent investigation. If two features had a Pearson correlation coefficient > 0.9 (21), the feature with the larger mean absolute coefficient was removed. Thirdly, the least absolute shrinkage and selection operator (LASSO) was used to select nonzero coefficient features associated with DMI in G 1/2-EEC patients with 10-fold cross-validation by the penalty parameter to avoid overfitting. The process of feature selection using the LASSO algorithm is shown in **Figure 2**. A radiomics signature was generated *via* a linear combination of selected features weighted by their respective coefficients.

### Radiomics Nomogram Development, Validation, and Diagnostic Performance

Based on the data of the primary group, the radiomics nomogram was developed using multivariable logistic regression by combining the radscore with the clinical parameters (CA125 and tumor size) selected and referred to other studies (22–24). The nomogram was then validated in the validation group.

All the MRI sequences (axial, sagittal, and/or coronal) were reviewed independently by the two radiologists who were available for preoperative clinical information, but blind to the results of surgical histopathology. MI was evaluated as the absence or superficial myometrial invasion (SMI) (MI < 50%) and DMI (MI ≥ 50%). To investigate the clinical application of the radiomics nomogram, after a period of 60 days, all cases were repeatedly reviewed by the same radiologists referring to the nomogram’s prediction possibility for DMI of each patient, the diagnoses were made, and the radiologists were named with the aid of nomogram.

To evaluate DMI in G 1/2-EEC for OPT, the diagnostic performances of the radiomics nomogram and the radiologists without and with the aid of the nomogram were assessed using receiver operating characteristic (ROC) curves in the primary and validation groups. The net reclassification index (NRI) and total integrated discrimination index (IDI) based on the whole data set of the radiologists without and with the aid of the nomogram were compared. Calibration curves were plotted using the Hosmer–Lemeshow (H-L) test to evaluate the calibration performance, which measured how close the prediction value generated by the nomogram was to the observed outcome. A significant result indicated a disagreement between the prediction value and the observed outcome of the nomogram.

### Statistical Analysis

The sample size for this study in the validation group was calculated as the following: if the study achieves 99% diagnostic performance (β = 0.01, α = 0.05) with mean nomogram scores of 0.6 and 0.1 in SMI and DMI patients, respectively, 115 samples are needed. If the study achieves 85%–95% diagnostic performance, 70 samples are needed. We included 105 patients finally.

All statistical analyses were performed using R software (Version 4.0.2; http://www.r-project.org). Student’s t-test was used to compare quantitative variables (age, tumor size, and CA125), and the Mann–Whitney U test, chi-squared test, or Fisher’s exact test was used to compare qualitative variables. The DeLong test was used to compare the diagnostic performance of the radiomics nomogram, the radiologists without and with the aid of the nomogram. The “rms” package, “pROC” package, “dca. R” package, and “PredictABEL” package were used for the analyses. The R code is supplied in the **Supplement Material**. A p value < 0.05 was considered statistically significant.

## Results

Finally, 209 G 1/2-EEC patients (mean 39 ± 4.3 years; range 25–45 years) were retrospectively included in the primary group. The clinicopathological characteristics of the included G 1/2-EEC patients are summarized in **Table 1**. The 209 EEC patients included 155 G1 and 54 G2 patients. The multivariate logistic regression analysis showed that the predictive factors for DMI in G 1/2-EEC were ER (with 0.75 of OR), PR (with 1.26 of OR), and CA125 (with 1.01 of OR), which are shown in **Table 2**.

**Table 1:** Clinicopathologic characteristics of the included grade 1/2 EEC patients.

	Primary group			Validation group		
	SMI	DMI	P value	SMI	DMI	p value
	(n = 83)	(n = 21)		(n = 87)	(n = 18)	
Age (year)	39.9 ± 4.6	41.4 ± 2.7	0.051	38.0 ± 4.2	42.3 ± 2.8	<0.001
CA125 (U/ml)	25.0 ± 20.3	47.9 ± 18.7	<0.001	16.4 ± 9.1	40.0 ± 29.3	0.003
Tumor size (mm)	44.9 ± 16.5	41.8 ± 16.4	0.437	46.5 ± 17.1	42.6 ± 10.7	0.220
Radscore	0.12 ± 0.2	0.52 ± 0.2	<0.001	0.17 ± 0.2	0.35 ± 0.2	0.006
ER (+/-)	53/30	6/15	0.008	59/28	7/11	0.041
CK7 (+/-)	50/33	10/11	0.685	76/11	12/6	0.069
PR (+/-)	41/42	15/6	0.118	63/24	9/9	0.113
Ki67 (+/-)	5/78	4/17	0.144	17/70	4/14	1.000
P53 (+/-)	40/43	12/9	0.625	63/24	8/10	0.042
Radiologist 1			<0.001			<0.001
SMI	78 (94.0%)	7 (33.3%)		85 (97.7%)	6 (33.3%)	
DMI	5 (6.0%)	14 (66.7%)		2 (2.3%)	12 (66.7%)	
Rad 1 + nomogram			<0.001			<0.001
SMI	83 (94.0%)	7 (33.3%)		86 (98.9%)	2 (11.1%)	
DMI	5 (6.0%)	14 (66.7%)		1 (1.1%)	16 (88.9%)	
Radiologist 2			<0.001			<0.001
SMI	79 (95.2%)	5 (23.8%)		86 (98.9%)	6 (33.3%)	
DMI	4 (4.8%)	16 (76.2%)		1 (1.1%)	12 (66.7%)	
Rad 2 + nomogram			<0.001			<0.001
SMI	83 (100%)	1 (4.8%)		87 (100%)	2 (11.1%)	
DMI	0 (0%)	20 (95.2%)		0 (0.0%)	16 (88.9%)	
Tumor grade			0.017			0.141
Grade 1	64 (77.1%)	10 (47.6%)		70 (80.5%)	11 (61.1%)	
Grade 2	19 (22.9%)	11 (52.4%)		17 (19.5%)	7 (38.9%)	

Continuous variables are presented as the mean ± standard deviation.

+/-, positive/negative; CA125, cancer antigen 125; DMI, deep myometrial invasion; EEC, endometrioid adenocarcinoma; FIGO, International Federation of Obstetrics and Gynecology; SD, standard deviation; SMI, superficial myometrial invasion; Rad, radiologist.

**Table 2:** The univariate and multivariate logistic regression analyses for independent predictive factors of DMI in patients for ovarian preservation.

Variables	Univariate analysis			Multivariate analysis		
	Odds ratio	95% CI	p-value	Odds ratio	95% CI	p-value
Age	1.01	1.00–1.03	0.141	1.01	0.99–1.02	0.274
CA125	1.01	1.00–1.01	<0.001	1.01	1.00–1.01	<0.001
Tumor size (mm)	1.00	0.99–1.00	0.435	1.00	1.00–1.00	0.706
ER	0.79	0.68–0.92	0.003	0.75	0.62–0.90	0.002
PR	1.15	0.99–1.35	0.072	1.26	1.06–1.50	0.011
CK7	0.95	0.81–1.11	0.518	1.06	0.86–1.30	0.586
Ki67	1.30	0.99–1.71	0.059	1.25	0.97–1.59	0.082
P53	1.06	0.91–1.24	0.469	1.04	0.88–1.25	0.629

CA125, cancer antigen 125; ER, estrogen receptor; PR, progesterone receptor.

### Feature Selection and Model Building

**Figure 3:**
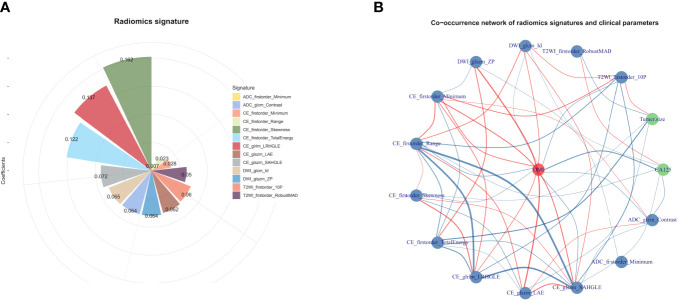
**(A)** The selected features for DMI in G 1/2-EEC patients for ovarian preservation. The chart shows that the features contribute to the radiomics signature with their coefficients obtained from linear regression. **(B)** The co-occurrence matrix plots the correlations of the patients for DMI (red spot), clinical parameters (CA125 and tumor size), and radiomics features. The blue line indicates a negative correlation, and the red line indicates a positive correlation (p < 0.05).

A total of 358 radiomics features, including 14 shape features, 72 first-order features, and 272 texture features, were extracted from the T2WI, DWI, ADC, and CE-T1WI sequences. Features with either interobserver or intraobserver ICC < 0.75 were removed, leaving 191 features. Based on the primary group, features with Pearson correlation coefficients > 0.9 were removed, leaving 160 features. After LASSO analysis, 13 radiomics features were finally included to form the radiomics signature. The 13 radiomics features including ADC_firstorder_Minimum and other features for assessing the depth of MI are shown in **Figure 3A**. The radscore calculation is shown in the following:

Radscore = 0.20192 + 0.05968×T2WI_firstorder_10P + -0.05049 × T2WI_firstorder_RobustMAD + 0.06464 × DWI_glcm_Id + -0.06397 × DWI_glszm_ZP + -0.0279 × CE_firstorder_Minimum + -0.02266 × CE_firstorder_Range + 0.16195 × CE_firstorder_Skewness + -0.12198 × CE_firstorder_TotalEnergy + 0.13675 × CE_glrlm_LRHGLE + -0.06215 × CE_glszm_LAE + 0.07236 × CE_glszm_SAHGLE + 0.00689 × ADC_firstorder_Minimum + 0.06399 × ADC_glcm_Contras

**Figure 4:**
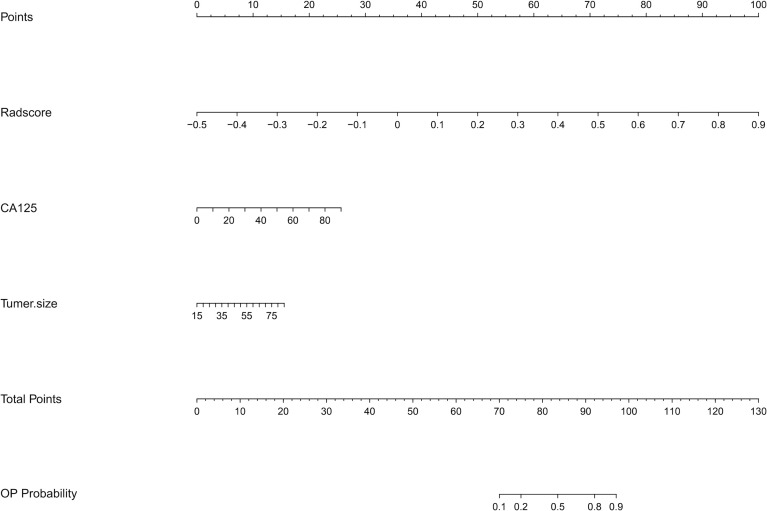
The radiomics nomogram incorporating the selected radiomics features with clinical parameters (CA125 and tumor size) in the primary group. To use the nomogram, locating the margin according to the patient information, drawing a line straight up to the point axis to obtain the score associated with the margin, and repeating for the radscore. By summing the scores of each point and locating it on the total points and drawing a line straight down to the bottom axis, the estimated probability of DMI in G 1/2-EEC patients for ovarian preservation can be determined.

**Figure 5:**
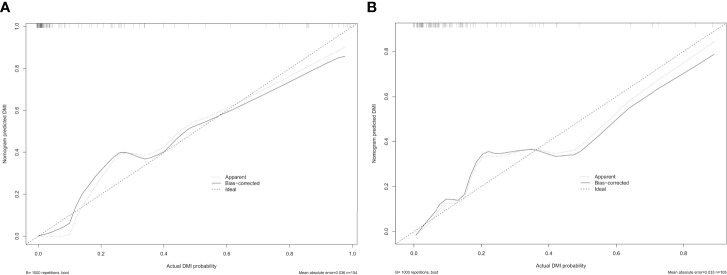
The calibration curve of the radiomics nomogram for predicting DMI in G 1/2-EEC patients, **(A)** in the primary group (p = 0.987) and **(B)** in the validation group (p = 0.437), which indicates the agreement between the prediction value and the observed outcome of the radiomics nomogram.

The co-occurrence network of the connection of each radiomics feature and clinical information are shown in **Figure 3B**. An MRI radiomics nomogram was further developed by incorporating the 13 radiomics features with CA125 and tumor size by linear regression to assess DMI in G 1/2-EEC in the primary group and is shown in **Figure 4**. Moreover, the calibration curves of the nomogram in the primary and validation groups are as shown in **Figure 5**.

### The Reason for Misjudgment

**Figure 6:**
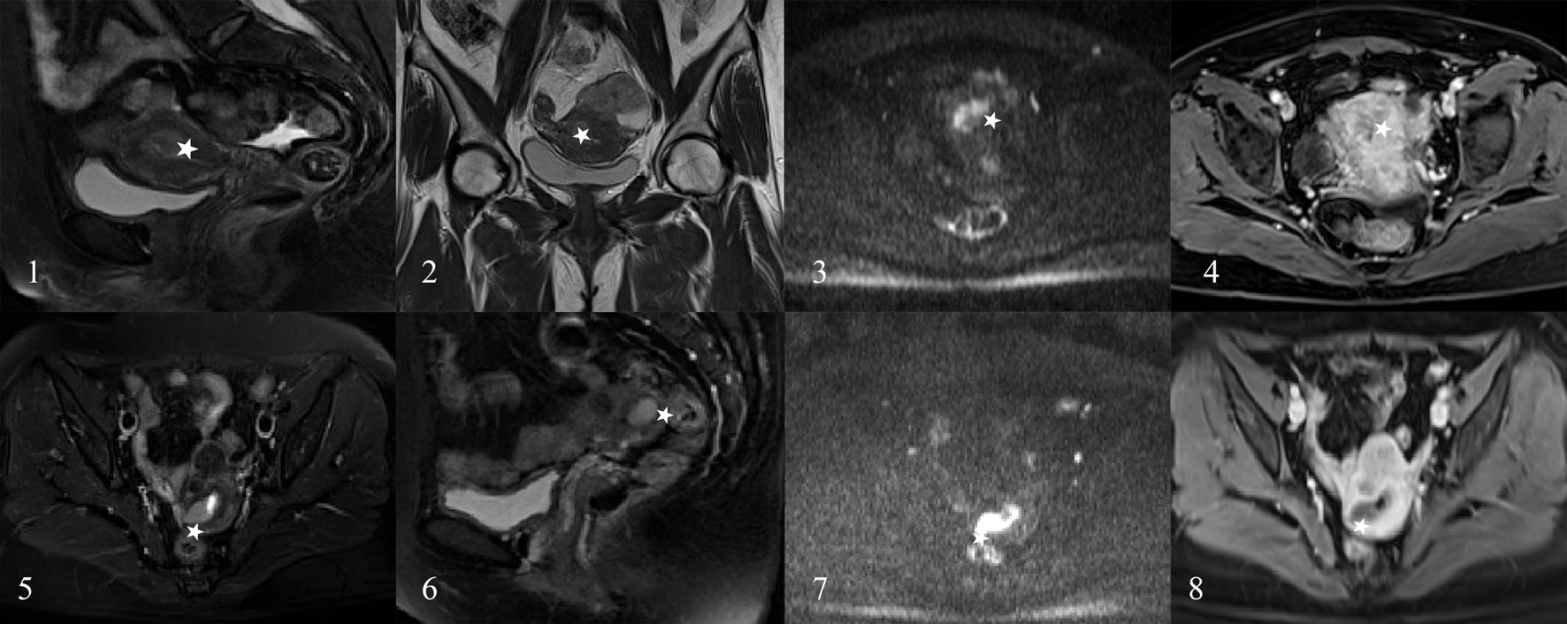
The EC patients misjudged by radiologists. A 45-year-old woman (images 1 to 4) and a 43-year-old woman (images 5 to 8) who were diagnosed as the DMI by radiologists (stars) and diagnosed as superficial myometrial invasion (SMI) by the radiomics nomogram and proven the SMI by pathology.

In the 209 EEC patients, 27 patients had adenomyosis, 70 patients had leiomyoma, and 22 patients had both adenomyosis and leiomyoma at the final pathological diagnosis. The misjudgment reasons and cases for the radiomics nomogram and radiologists are exhibited in **Table 3**. The radiomics nomogram misjudged the depth of MI mostly due to the small tumor size, which indicated that the drawing of ROI might influence the diagnostic performance (**Figure 6**).

**Table 3:** The certain cases of misjudgment with the reason in the radiomics nomogram and radiologists.

	Misjudgment	Adenomyosis	Leiomyoma	Cornua uteri	Small tumor size
Radiomics nomogram	SMI	2	2	2	15
	DMI	1	4	0	0
Radiologist 1	SMI	2	1	3	0
	DMI	6	6	1	0
Radiologist 2	SMI	2	1	2	0
	DMI	5	5	1	0

DMI, deep myometrial invasion; SMI, superficial myometrial invasion.

### Diagnostic Performance Assessment

**Figure 7:**
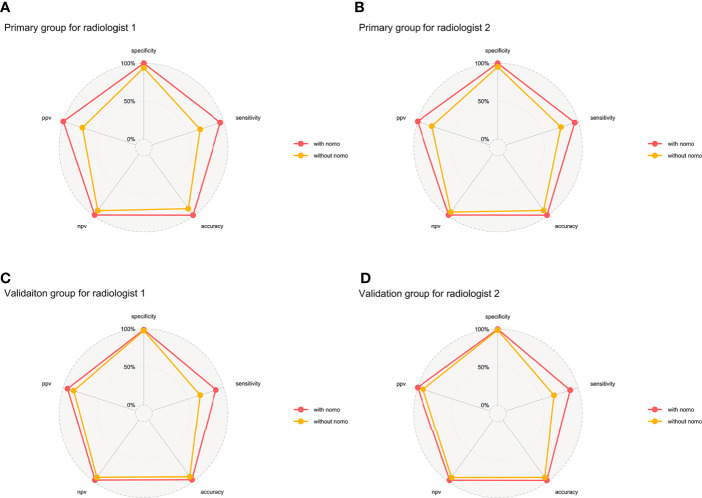
Binary diagnosis of the two radiologists without and with the aid of a nomogram. **(A, B)** are for the primary group; **(C, D)** are for the validation group. PPV, positive predictive value; NPV, negative predictive value.

The AUC, sensitivity, specificity, accuracy, and negative and positive predictive values (NPV and PPV) for the radiologists without and with the aid of the nomogram in the primary and validation groups for identifying DMI in G 1/2-EEC patients for OPT are shown in **Table 4** and **Figure 7**. For identifying DMI in G 1/2-EEC, ROC curve analyses showed that the AUCs were 0.92 and 0.70 for the radiomics signature; were 0.96 and 0.88 for the radiomics nomogram in the primary and validation groups, respectively; were 0.80, 0.86, 0.98, and 0.98 for radiologists 1 and 2 without and with the nomogram aid in the primary group; and were 0.82, 0.83, 0.94, and 0.94 in the validation group, respectively. The DeLong test showed that the AUCs of the radiomics nomogram were higher than that of radiologist 1 (p = 0.009), but not in the primary group of radiologist 2 (p = 0.061) and radiologists 1 and 2 in the validation group (p = 0.405 and 0.450, respectively). The AUC of the radiologists with the aid of the nomogram was higher than those of radiologists 1 and 2 alone in the primary (p < 0.001 and p = 0.009) and validation groups (p = 0.023 and 0.021). The AUCs of each selected clinical characteristic and radiomics feature are shown in the **Supplementary Table**.

**Table 4:** The diagnostic performance of the radiologists without and with the aid of the nomogram in the primary and validation groups.

Model	AUC	p value*^c^*	SEN	SPE	ACC	NPV	PPV
	(95% CI)						
**Primary group**							
Radiomics	0.96 (0.92–0.99)	0.009*^a^*	100%	83.15%	86.5%	1.00	0.60
nomogram		0.061*^b^*					
Radiologist 1	0.80 (0.70–0.91)		66.7%	94.0%	88.5%	0.92	0.74
Radiologist 1	0.98 (0.93–1.00)	<0.001*^c^*	95.2%	100%	99.0%	0.99	1.00
+ nomogram							
Radiologist 2	0.86 (0.76–0.95)		76.2%	95.2%	91.3%	0.94	0.80
Radiologist 2	0.98 (0.93–1.00)	0.009*^c^*	95.2%	100%	99.0%	0.99	1.00
+ nomogram							
**Validation group**							
Radiomics	0.88 (0.80–0.96)	0.405*^a^*	72.2%	89.6%	86.7%	0.94	0.59
nomogram		0.450*^b^*					
Radiologist 1	0.82 (0.71–0.93)		66.7%	97.7%	92.4%	0.93	0.86
Radiologist 1	0.94 (0.86–1.00)	0.023*^c^*	88.9%	98.8%	97.1%	0.98	0.94
+ nomogram							
Radiologist 2	0.83 (0.72–0.94)		66.7%	98.8%	93.3%	0.93	0.92
Radiologist 2	0.94 (0.87–1.00)	0.021*^c^*	88.9%	100%	98.1%	0.98	1.00
+ nomogram							

ACC, accuracy; AUC, area under the curve; CI, confidence interval; SEN, sensitivity; SPE, specificity; NPV, negative predictive value; PPV, positive predictive value.

a vs. radiologist 1.

b vs. radiologist 2.

c Without vs. with the aid of the nomogram.

**Figure 8:**
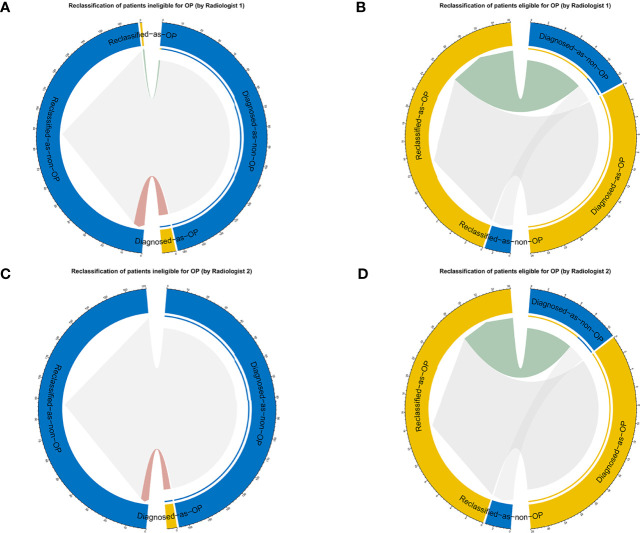
The reclassification results are shown as circle plots. Reclassification of patients for different groups (**A, B** for radiologist 1, and **C, D** for radiologist 2). Groups were illustrated according to the molder of the radiologists with the aid of a nomogram. OP, ovarian preservation.

The reclassification measures of discrimination confirmed that the radiologists with the aid of the nomogram performed better than the radiologists alone based on the whole data, with an NRI of 0.29 (95% CI: 0.15–0.43) and an IDI of 0.43 (95% CI: 0.32–0.54) for radiologist 1 and with an NRI of 0.23 (95% CI: 0.10–0.36) and an IDI of 0.37 (95% CI: 0.26–0.47) for radiologist 2 (both p < 0.01) (**Figure 8**). These results indicated that 37–43 patients per 100 patients would have an accurate assessment of DMI using the MRI radiomics nomogram.

## Discussion

In this study, a radiomics nomogram was developed by combining multiparametric MRI radiomics features and clinical information to assess the depth of MI in G1/2-EEC patients to select eligible OPT patients. The radiomics nomogram could aid radiologists in making decisions in selecting eligible OPT patients by assessing MI. NRI and IDI analyses showed a better clinical usefulness in the radiologists with the aid of the nomogram than in the radiologists alone for individually identifying MI in choosing eligible OPT patients.

In addition to the immediate consequences of hot flashes and vaginal atrophy, BSO causes surgical menopause in young women, which results in a number of long-term sequelae, including an increased risk of cardiovascular disease, osteoporosis, hip fracture, and cognitive dysfunction (25, 26). A meta-analysis showed that the relative risk of cardiovascular disease was 2.62 in women who underwent BSO (25). In young women, BSO alone tended to increase the risk of myocardial infarction with a relative risk of 1.6 (27). A prospective, population-based cohort study found that women who underwent prophylactic BSO before the age of 45 had a 67% increase in mortality, mainly in women who had not received estrogen treatment (28). Ovarian conservation had no effect on either cancer-specific or overall survival (6, 7). Therefore, to avoid the adverse consequences of BSO, there is a strong rationale for OPT in young early-stage EC patients.

Early-stage EC patients younger than 45 are recommended to receive OPT. The assessment of the depth of MI may be a challenge when 1) the tumor involves the uterine cornu; 2) the uterine anatomy is distorted by leiomyomas or adenomyosis; 3) a large endometrial tumor distends and thins the myometrium; and 4) the endometrial tumor is relatively isointense to the myometrium on T2WI. In these clinical scenarios, radiologists have difficulty assessing MI properly and should be aware of possible tumor overstaging (29). Under these circumstances, radiomics nomograms may be a particularly useful tool to improve the delineation of tumor margins and to avoid overestimation of tumor extent, as shown in this study.

Recently, a computerized deep-learning model was developed to automatically evaluate the depth of MI in EC patients, with a sensitivity of 66.6% and a specificity of 87.5%. The results showed a better and more time-efficient performance in the deep learning model than the radiologists (14). In contrast, our radiomics nomogram model combined MRI-based radiomics features and the clinical characteristics (tumor size and CA125) to yield a high accuracy in assessing the DMI of early-stage EC patients. The radiomics nomogram could generate a certain possibility of DMI for each patient to help radiologists assess the depth of MI.

Some studies have suggested that MRI-based texture analysis could be helpful in determining the depth of MI in EC patients (13, 30). Ytre-Hauge et al. obtained an accuracy of 78% for DMI detection, which was significantly higher than that of the radiologists reading (accuracy of 70%) in the same population (30). A recent study used MRI radiomics-powered machine learning to help radiologists evaluate the presence of DMI, yielding an accuracy of 86% and an AUC of 0.92 and increasing the radiologists’ performance from 82% to 100% (p = 0.48). However, this study included only 54 patients and extracted features only from T2WI (31).

In our study, the multiparametric MRI radiomics nomogram was generated, which contributed to good diagnostic performance by unraveling more comprehensive information about tumor heterogeneity. The difference between our study and previous studies was that our nomogram was used to offer the radiologists a certain possibility of DMI and gave them a hint of the depth of MI. When radiologists face specific perplexing clinical scenarios, this radiomics nomogram could help them obtain a more confident diagnosis. Furthermore, the reclassification framework was used to provide an outcome prediction analysis of clinical decision-making. The clinical benefits were significantly improved, with IDIs of 0.43 and 0.37 in radiologists 1 and 2, respectively, which indicated that 37–43 out of 100 patients would be reclassified correctly from the radiomics-aided radiologists’ prediction compared to the radiologists alone.

There were some limitations in our study. First, we did not include high-order wavelet features because previous studies suggested that wavelet features were not stable and lacked reasonable clinical interpretation (32). Second, this was a single-center scanner study; therefore, our results should be validated on data from multiple centers and from different scanners prior to clinical implementation. Third, the deep-learning based features were not investigated although some studies of other cancers showed a good performance (33–35). Last, the imbalance of the DMI and SMI datasets was not balanced by using any techniques such as the synthetic minority oversampling technique; however, we tried to select robust and reproducible features with a stable diagnostic performance.

In conclusion, the multiparametric MRI-based radiomics nomogram outperformed the radiologists in assessing the depth of MI in G 1/2-EEC patients younger than 45 years and for selecting eligible OPT patients. It could help radiologists significantly improve the predictive performance of MI status.

## Data Availability Statement

The raw data supporting the conclusions of this article will be made available by the authors, without undue reservation.

## Ethics Statement

The studies involving human participants were reviewed and approved by the ethics committee of Obstetrics & Gynecology Hospital, Fudan University. The patients/participants provided their written informed consent to participate in this study.

## Author Contributions

The study supervisor is JQ. The authors responsible for the study concept, design, and implementation are BY and XM. YL contributed to the data analysis and performed the statistical analysis. SD helped in giving the R code. BY, XM, and GZ contributed to the data acquisition. BY and YL conducted the ROI drawing. BY was the major contributor and contributed to writing the first draft. JQ revised and edited the manuscript. All authors contributed to the article and approved the submitted version.

## Funding

This study was supported by the National Natural Science Foundation of China (No. 81971579) and Shanghai Municipal Health Commission (No. ZK2019B01).

## Conflict of Interest

Author SD was employed by company GE Healthcare.

The remaining authors declare that the research was conducted in the absence of any commercial or financial relationships that could be construed as a potential conflict of interest.

The reviewer YR declared a shared affiliation with several of the authors BY, XM, YL, GZ, and JQ to the handling editor at the time of the review.

## Publisher’s Note

All claims expressed in this article are solely those of the authors and do not necessarily represent those of their affiliated organizations, or those of the publisher, the editors and the reviewers. Any product that may be evaluated in this article, or claim that may be made by its manufacturer, is not guaranteed or endorsed by the publisher.
