# Supplementary material for: MRI-Based Radiomics Nomogram for Selecting Ovarian Preservation Treatment in Patients With Early-Stage Endometrial Cancer
Source: Front Oncol. 2021 Sep 9;11:730281. doi: 10.3389/fonc.2021.730281 (PMC8459685; doi:10.3389/fonc.2021.730281)
Supplement: Supplementary file 1 [file DataSheet_1.docx]

Supplement Table. The AUCs of the radiomics features and clinical characteristics.

| Features | AUC | 95% confidence interval |
| --- | --- | --- |
| T2WI_firstorder_10P | 0.66 | 0.52-0.80 |
| T2WI_firstorder_RobustMAD | 0.62 | 0.48-0.75 |
| DWI_glcm_Id | 0.60 | 0.47-0.73 |
| DWI_glszm_ZP | 0.61 | 0.48-0.74 |
| CE_firstorder_Minimum | 0.77 | 0.65-0.88 |
| CE_firstorder_Range | 0.68 | 0.54-0.83 |
| CE_firstorder_Skewness | 0.63 | 0.48-0.78 |
| CE_firstorder_TotalEnergy | 0.59 | 0.47-0.72 |
| CE_glrlm_LRHGLE | 0.57 | 0.42-0.72 |
| CE_glszm_LAE | 0.50 | 0.36-0.64 |
| CE_glszm_SAHGLE | 0.68 | 0.53-0.82 |
| ADC_firstorder_Minimum | 0.53 | 0.40-0.66 |
| ADC_glcm_Contrast | 0.65 | 0.53-0.77 |
| CA125 | 0.83 | 0.76-0.91 |
| Tumor size | 0.56 | 0.41-0.70 |

Supplement files of R code

sink("result.txt")

library(dplyr);library(readxl);library(irr);library(caret);library(rms);library(glmnet);library(pROC);library(PredictABEL)

ICC.data <- read_excel("./data.xlsx",sheet = "icc")

intra.icc.data <- subset(ICC.data,ICC.data[,1] != 3)[,-(1:2)]

inter.icc.data <- subset(ICC.data,ICC.data[,1] != 2)[,-(1:2)]

ICC <- function(x){icc(cbind(x[1:50],x[51:100]))$value}

icc.intra <- data.frame(apply(intra.icc.data, 2, ICC))

intra.icc <- rownames(subset(abs(icc.intra), icc.intra > 0.75 ))

icc.inter <- data.frame(apply(inter.icc.data, 2, ICC))

inter.icc <- rownames(subset(abs(icc.inter), icc.inter > 0.75 ))

ICCfeature <- intersect(intra.icc,inter.icc)

new <- read_excel("./data.xlsx",sheet = "nri")

data1 <- data.frame(y = new$HMI, new$Primaryold)

data2 <- data.frame(y = new$HMI, new$Primarynew)

data3 <- data.frame(y = new$HMI, new$Advancedold)

data4 <- data.frame(y = new$HMI, new$Advancednew)

model1 <- glm(y~.,data = data1,x = T,y = T,family = binomial('logit'))

model2 <- glm(y~.,data = data2,x = T,y = T,family = binomial('logit'))

model3 <- glm(y~.,data = data3,x = T,y = T,family = binomial('logit'))

model4 <- glm(y~.,data = data4,x = T,y = T,family = binomial('logit'))

old1 <- model1$fitted.values

new1 <- model2$fitted.values

old2 <- model3$fitted.values

new2 <- model4$fitted.values

reclassification(data = data1,cOutcome = 1,predrisk1 = old1,predrisk2 = new1,cutoff = c(0,0.5,1))

reclassification(data = data3,cOutcome = 1,predrisk1 = old2,predrisk2 = new2,cutoff = c(0,0.5,1))

set.seed(666)

excel <- read_excel("./data.xlsx",sheet = "data")

data <- read_excel("./data.xlsx",sheet = "val")

DATA <- excel

clin.data.p <- data.frame(y=DATA$HMI ,CA125 = DATA$CA125, Tumer.size = DATA$`Tumor Size`)

clin.model.p <- glm(y~.,data = clin.data.p)

clinscore.p <- predict(clin.model.p)

clin.data.v <- data.frame(y=data$HMI,CA125 = data$CA125, Tumer.size = data$`Tumor Size`)

clin.model.v <- glm(y~.,data = clin.data.v)

clinscore.v <- predict(clin.model.v)

icc.excel <- excel[ICCfeature]

ttest<- function(x){t.test(x, excel$HMI)$p.value}

ttest.p <- apply(icc.excel,2,ttest)

icc.index<-which(ttest.p < 0.001)

iccfeature <- ICCfeature[icc.index]

icc.data <- DATA[iccfeature]

p.data <- preProcess(icc.data ,method = c("center","scale","corr"),cutoff = 0.99)

P.data <- predict(p.data,newdata = icc.data)

lasso.x <- as.matrix(P.data);lasso.y <- as.matrix(DATA$HMI)

lasso.cv <- cv.glmnet(x = lasso.x,y = lasso.y,family = "binomial",type.measure = "auc")

lasso <- glmnet(x = lasso.x,y = lasso.y,family = "binomial")

number <- which(coef(lasso,s = lasso.cv$lambda.min) != 0)

lasso.coef <- coef(lasso,s = lasso.cv$lambda.min)[number][-1]

signature <- colnames(P.data[number - 1])

print(signature)

train <- data.frame(HMI=DATA$HMI,CA125=DATA$CA125,ER=DATA$ER,Age=DATA$Age,Tumer.size=DATA$`Tumor Size`,P.data[signature])

v.data <- preProcess(data[signature],method = c("center","scale"))

V.data <- predict(v.data,newdata = data[signature])

test <- data.frame(HMI=data$HMI,CA125=data$CA125,ER=data$ER,Age=data$Age,Tumer.size=data$`Tumor Size`,V.data)

rad.model.p <- glm(y~.,data = data.frame(y = train$HMI, train[signature]))

radscore.p <- predict(rad.model.p) #radscore

radscore.v <- predict(rad.model.p, newdata = data.frame(y = test$HMI, test[signature]))

rad.coef <- round(rad.model.p$coefficient[-1],5)

equation <- round(rad.model.p$coefficient[1],5)

for (n in 1:length(signature))

{ equation <- paste(equation," + ",rad.coef[n],"×",signature[n],sep = "") }

print(paste("Radscore = ",equation))

nomo.data.p <- data.frame(HMI = train$HMI, Radscore = radscore.p,CA125 = train$CA125, Tumer.size = train$Tumer.size)

dd = datadist(nomo.data.p) ; options(datadist = "dd")

nomo.model.p <- lrm(HMI~.,data = nomo.data.p,x = T,y = T)

nomoscore.p <- predict(nomo.model.p)

nomo.data.v <- data.frame(HMI = test$HMI, Radscore = radscore.v, CA125 = test$CA125, Tumer.size = test$Tumer.size)

dd = datadist(nomo.data.v);options(datadist = "dd")

nomo.model.v <- lrm(HMI~.,data = nomo.data.v,x = T,y = T)

nomoscore.v <- predict(nomo.model.v,newdata= nomo.data.v)

nomo.pic <- nomogram(nomo.model.p, fun.at=c(0.1,0.2,0.5,0.8,0.9),fun = function(x)1/(1 + exp(-x)),lp = F)

plot(nomo.pic)

#-----------------------roc-----------------------------------

auc1 <- roc(response=DATA$HMI,predictor=DATA$Primaryold,ci = T, ci.alpha = 0.9,percent = T);auc1;pROC::coords(auc1, "best",ret="all", transpose=F)

auc11 <- roc(response=DATA$HMI,predictor=as.numeric(DATA$Primarynew),ci = T, ci.alpha = 0.9,percent = T);auc11;pROC::coords(auc11, "best",ret="all", transpose=F)

auc2 <- roc(DATA$HMI,DATA$Advancedold,ci = T, ci.alpha = 0.9,percent = T);auc2;pROC::coords(auc2, "best",ret="all", transpose=F)

auc22 <- roc(DATA$HMI,DATA$Advancednew,ci = T, ci.alpha = 0.9,percent = T);auc22;pROC::coords(auc22, "best",ret="all", transpose=F)

auc3 <- roc(train$HMI,nomoscore.p,ci = T, ci.alpha = 0.9,percent = T);auc3;pROC::coords(auc3, "best",ret="all", transpose=F)

auc33 <- roc(train$HMI,nomoscore.p,ci = T, ci.alpha = 0.9,percent = T);auc33;pROC::coords(auc3, "best",ret="all", transpose=F)

auc4 <- roc(test$HMI,nomoscore.v,ci = T, ci.alpha = 0.9,percent = T);auc4;pROC::coords(auc4, "best",ret="all", transpose=F)

auc44 <- roc(test$HMI,nomoscore.v,ci = T, ci.alpha = 0.9,percent = T);auc44;pROC::coords(auc4, "best",ret="all", transpose=F)

auc5 <-roc(data$HMI,data$Primaryold,ci = T, ci.alpha = 0.9,percent = T);auc5;pROC::coords(auc5, "best",ret="all", transpose=F)

auc55 <-roc(data$HMI,data$Primarynew,ci = T, ci.alpha = 0.9,percent = T);auc55;pROC::coords(auc55, "best",ret="all", transpose=F)

auc6 <-roc(data$HMI,data$Advancedold,ci = T, ci.alpha = 0.9,percent = T);auc6;pROC::coords(auc6, "best",ret="all", transpose=F)

auc66 <-roc(data$HMI,data$Advancednew,ci = T, ci.alpha = 0.9,percent = T);auc66;pROC::coords(auc66, "best",ret="all", transpose=F)

rad.p.auc <- roc(train[,1],radscore.p,ci = T, ci.alpha = 0.9,percent = T);rad.p.auc;pROC::coords(rad.p.auc, "best",ret="all", transpose=F)

rad.v.auc <- roc(test[,1], radscore.v,ci = T, ci.alpha = 0.9,percent = T);rad.v.auc;pROC::coords(rad.v.auc, "best",ret="all", transpose=F)

for (n in 1:length(signature)){print(signature[n]);auc <- roc(train$HMI, as.matrix(train[signature[n]]),ci = T, ci.alpha = 0.9,percent = T);print(auc)}

roc.test(auc1,auc11);roc.test(auc2,auc22);roc.test(auc3,auc33);roc.test(auc4,auc44);roc.test(auc5,auc55);roc.test(auc6,auc66)

roc.test(auc3,auc1);roc.test(auc3,auc11);roc.test(auc3,auc2);roc.test(auc3,auc22)

roc.test(auc4,auc5);roc.test(auc4,auc55);roc.test(auc4,auc6);roc.test(auc4,auc66)

roc.test(rad.p.auc,auc3);roc.test(rad.v.auc,auc4)

sink()
